# Do capability and functioning differ? A study of U.K. survey responses

**DOI:** 10.1002/hec.3586

**Published:** 2017-09-24

**Authors:** Hareth Al‐Janabi

**Affiliations:** ^1^ Health Economics Unit, Institute of Applied Health Research University of Birmingham Birmingham UK

**Keywords:** capability approach, ICECAP, outcome valuation, well‐being

## Abstract

A core feature of the capability approach is that a person's capabilities (what they are able to do and be in their life) can differ from their functionings (what they actually do and are in their life). However, the degree to which capability and functioning differ in practice is unclear. This paper investigates this issue, focusing on capability and functioning differences (CFD) across different aspects of life and different individuals. In the study, the ICECAP‐A capability questionnaire was modified to measure both functionings and capabilities and was completed by U.K.‐based convenience sample of 943 people. Around one third of people reported CFD in at least one area of their life, most commonly in terms of their “achievement.” People were more likely to report CFD when they had a degree‐level education and when they had impaired health. An additional finding was that capability varied more with education whereas functioning varied more with health status. This finding needs further examination, but it suggests that the choice of evaluative space may influence how priorities are set for public spending.

## 1 INTRODUCTION

There is substantial debate about how best to measure and value the benefit of public services and, in particular, health care interventions (Birch & Donaldson, 2003; Brazier, Ratcliffe, Salomon, & Tsuchiya, 2007; Brouwer, Culyer, Van Exel, & Rutten, 2008; Coast, Smith, & Lorgelly, 2008; Kahneman & Sugden, 2005). In the health sector, there is a growing sense that narrow measures of health gain may not be sufficient for evaluating the benefits of social care (Ryan, Netten, Skatun, & Smith, 2006), care for older people (Grewal et al., 2006), and mental health care (Simon et al., 2013). This has led to interest in operationalising Sen's capability approach. Capturing a person's capabilities is potentially difficult. It requires people to evaluate their “real opportunity” to achieve the things in life that they have reason to value (Sen, 2009). Most people seem able to report their capability level in various areas of their life (Al‐Janabi, Keeley, Mitchell, & Coast, 2013). But in doing so, it is unclear whether they differentiate what they are able to do (their “capability”) from what they actually do (their “functioning”). The assumption that capability and functioning can differ lies at the very heart of the capability approach (Bleichrodt & Quiggin, 2013); the focus of this paper is to study the extent to which reported capability and functioning actually differ in different areas of life.

### 1.1 The distinction between capability and functioning in theory

Capabilities relate to aspects of a person's life that they “have reason to value” (Sen, 1993). This can cover basic aspects of life such as the ability to have food and shelter, through to more complex aspects such as the ability to be socially integrated (Sen, 1993). Nussbaum advocates a core list of 10 central capabilities that cover broad terrain and include, for example, the capability for “bodily health,” “reasoning,” and “play”—capabilities she argues that all governments should aim to secure (Nussbaum, 2000). In each aspect of life, a person's capability may be associated with several different levels of functionings. So, for example, people who are equally capable of reasoning may exercise reasoning in their life to different degrees. Thus, in theory, a person's capability for reasoning may differ from their functioning and people with similar capabilities may exercise different levels of functionings.

There are a number of reasons for using capability information as opposed to functioning information when evaluating public interventions. First, capabilities may reveal important differences in well‐being (Sen, 2009). For example, a starving person and a fasting person may achieve similar levels of nutritional functioning, yet most people would conclude that the fasting person was better off than the starving person and it is this person's capability for nutrition that reveals this. Second, a person's capability set contains information on all functionings obtainable, thus it is argued that there is no loss in using capability information, relative to functioning information (Sen, 2009). Third, capabilities are arguably more appropriate for political goals because functionings cannot be directly created by public policy. For example, a policymaker cannot force good health (Anand, 2005), play, or love (Nussbaum, 2000); policymakers can only create opportunities for these functionings to be realised. Fourth, a focus on expanding capabilities enables people to impress their character on their own life and develop judgement and self‐control (Sugden, 2003).

Nussbaum highlights particular areas of life where people may rationally choose to reject higher functionings. For example, a person may reject leisure to dedicate more time to their work. Some religious people may reject nutrition to observe periods of fasting, or reject sex and reproduction to pursue a life of celibacy (Nussbaum, 2000). The selection of functionings from within a capability “set” can be characterised as a constrained choice, influenced by the person's preferences in tandem with social influences and personal history (Robeyns, 2005). Although there are prominent examples where capability and functioning may differ, these are quite specific and it is not clear whether capabilities and functionings are perceived to differ across fundamental aspects of life.

### 1.2 The measurement of capabilities

Until recently, most efforts to measure capabilities have focused on measuring functionings, as proxies for capabilities (Chiappero‐Martinetti & Roche, 2009; Cookson, 2005). In empirical studies, for example, survey data on whether the person has been a victim of an assault or whether they use their imagination are taken as an indicator of a person's capability to avoid bodily harm and capability for reasoning respectively (Anand et al., 2009). Indeed, some authors are sceptical that a person's capabilities can be directly captured at all. Doubts centre on the difficulty people would have in knowing what they are capable of (Gasper, 2007) and also on the fact that imposing some conceptual structure on people's opportunities in itself restricts what individuals might wish to choose (Sugden, 2003).

Nevertheless, interest has grown in health economics in developing measures to capture people's self‐reported capabilities. These self‐report measures have focused on capabilities relevant to older people (Coast, Flynn, et al., 2008), women (Greco, Skordis‐Worrall, Mkandawire, & Mills, 2015), and the general adult population (Al‐Janabi, Flynn, & Coast, 2012), and on measures of capability for evaluating interventions in public health (Lorgelly, Lorimer, Fenwick, Briggs, & Anand, 2015), social care (Burge, Netten, & Gallo, 2010), mental health (Simon et al., 2013), pain (Kinghorn, Robinson, & Smith, 2015), and end‐of‐life care (Canaway, Al‐Janabi, Kinghorn, Bailey, & Coast, 2017; Sutton & Coast, 2014). Arguably, the most well‐developed capability measures in terms of their testing and application in the health sector are the ICECAP capability measures (see http://www.icecap.bham.ac.uk).

Capability‐based outcome measures appear to perform well as indicators of well‐being (Al‐Janabi, Peters, et al., 2013; Malley et al., 2012), but it is unclear whether they capture capability information that is distinct from functioning information. Such information is necessary in understanding the value of capability‐based questions.

The objective of this study was to determine, quantitatively, the degree to which functionings and capabilities are perceived to differ. Capabilities and functionings were captured simultaneously using a modified survey (ICECAP‐A) tool administered to a convenience sample of adults in the U.K. The specific focus in this study is on the extent to which capability and functioning differences (which are referred to as “CFD” in this paper) varied across aspects of life and across respondents. The following section describes the survey instrument, data collection, and methods of analysis. The results and implications of the study are presented in the final two sections.

## 2 METHODS

### 2.1 Design of the survey instrument

**Figure 1:**
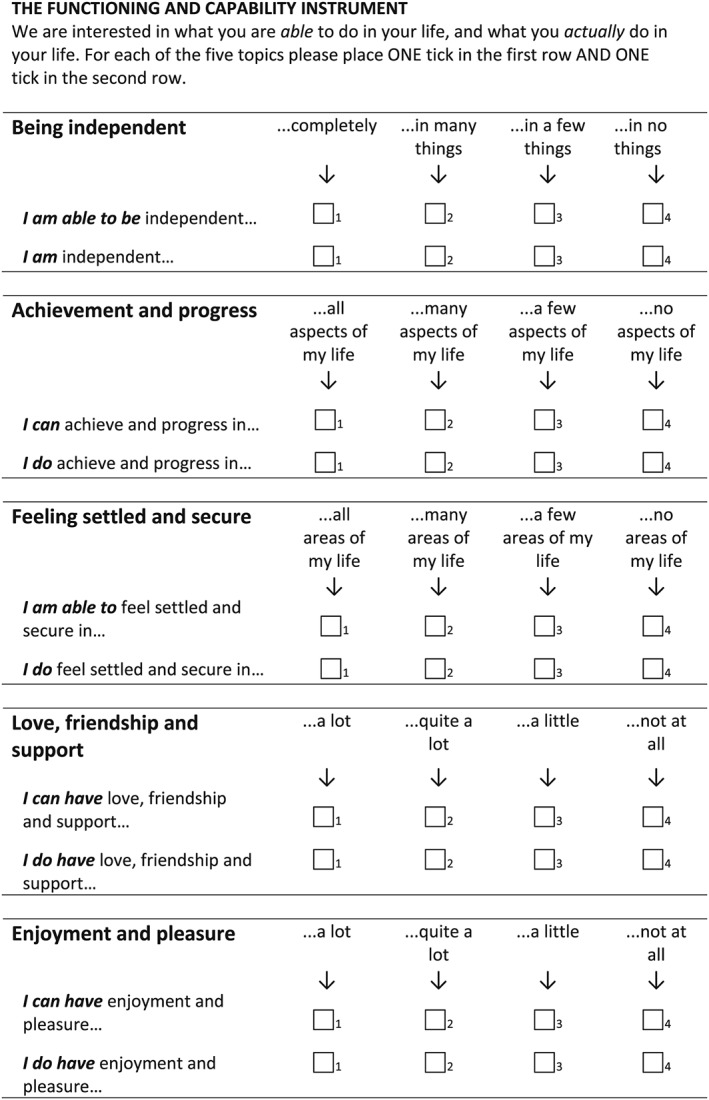
The functioning and capability instrument

The ICECAP‐A capability measure, developed for the general adult population, comprises questions about five attributes of a person's life: stability, attachment, autonomy, achievement, and enjoyment (Al‐Janabi et al., 2012). For each attribute, people report their level of capability from four choices (ranging from full capability to no capability—please see Figure 1 for more details). The focus in the ICECAP‐A is on assessing capability across a set of domains that were found to underpin well‐being for U.K. adults. A range of studies indicate that the ICECAP‐A measure is a broadly reliable, valid, and responsive measure of a person's well‐being (Al‐Janabi, Flynn, Peters, Bryan, & Coast, 2015; Al‐Janabi, Peters, et al., 2013; Goranitis, Coast, Al‐Janabi, Latthe, & Roberts, 2016; Keeley et al., 2015; Mitchell, Al‐Janabi, Richardson, Iezzi, & Coast, 2015).

For this study, the ICECAP‐A measure was redesigned to assess both capabilities and functionings (see Figure 1). This comprised an explicit statement that respondents report what they were *able* to do and what they *actually* do in each area of their life. The revised instrument included two response rows per attribute for people to report their level of capability and their level of functioning. An additional revision was a reordering of the items in view of the perceived difficulty that respondents might have in distinguishing capability from functioning on the initial attribute (capability to feel settled and secure).

**Figure 2:**
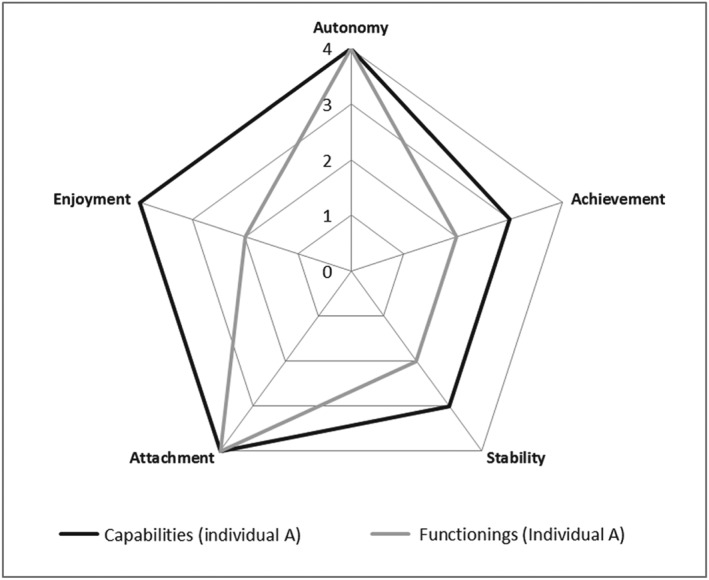
Radar plot of person A's capability set and functionings, where 4 represents maximum capability/functioning and 1 represents no capability/functioning

Capability and functioning responses to the amended ICECAP measure can be plotted as a capability set encasing chosen functionings. Figure 2 shows a person reporting Level 4 (top level) capability in autonomy, enjoyment, and attachment, and Level 3 capability in stability and achievement. In terms of functionings, this person reports Level 4 autonomy and attachment and Level 2 stability, enjoyment, and achievement.

### 2.2 Data collection

Data were collected on capability and functioning responses, by including the modified functioning and capability instrument opportunistically in a survey about the impact of illness on the family. The instrument was nested into a 14‐page follow‐up questionnaire, sent to all people (*n* = 1,627) who returned a baseline questionnaire in 2012 or were involved in the pilot study (Al‐Janabi et al., 2016). The follow‐up questionnaire was completed by family members of people with long‐term after‐effects of meningitis—a predominantly childhood illness that can have long‐term impacts on the survivors and their families. The questionnaire covered (a) the health status of the patient; (b) the social network of the patient; (c) informal care provided by the family member; (d) the health status and well‐being of the family member; and (e) the capabilities and functionings of the family member. Socio‐demographic data were collected in the baseline questionnaire. The focus in this study is on the capabilities and functionings data and how responses varied across respondents with different characteristics.

Questionnaires were included in a survey pack with information about the study and posted to 1,627 individuals in May 2013. A reminder postcard was sent to all individuals after 1 week, and a reminder letter was sent to all nonresponders after 4 weeks. Respondents were excluded if the family network broke down between baseline and follow‐up or if the questionnaire was completed incorrectly (for example, by the wrong family member). The study protocol was approved by the University of Birmingham's Life and Health Sciences Ethical Review Committee (ERN_11‐0191).

### 2.3 Measures in the survey

Data were collected on a number of variables that may influence individuals' reported capability. These variables were as follows:

- *Health status*: This was measured using the EQ‐5D‐5L (Herdman et al., 2011). This is a generic health status measure advocated by the U.K. National Institute for Health and Care Excellence for calculating quality‐adjusted life years (NICE, 2013). The EQ‐5D‐5L assesses an individual's health status across five items (mobility, self‐care, usual activities, pain/discomfort, and anxiety/depression). Each item has five response options ranging from no impairment to extreme impairment. An index score, where 0 is state equal to death and 1 is full health, was generated for all respondents using the interim U.K. scoring algorithm (Van Hout et al., 2012).
- *Education level*: Education was assessed in terms of the respondent's highest level of educational or technical qualification, with the options being no qualifications/General Certificate of Secondary Education (GCSE) (typically for 16‐year‐olds) or equivalent/A‐level (typically for 18‐year‐olds) or equivalent/degree.
- *Caring responsibility*: This was assessed in terms of whether the respondent reported any informal care tasks (personal care, organisational support, and additional household activity) for the person who had survived meningitis.

### 2.4 Analysis

The analysis was based on the data of all individuals who completed a valid segment of the functioning and capability instrument, unless the specific analysis (e.g., reporting the number of CFDs across the questionnaire) requires full completion of the instrument. Differences between capability and functioning responses (CFD) were studied at the attribute level. This means that people who fully completed the ICECAP‐FC would generate five functioning and capability responses, or “segments.” Analysis was conducted to determine the proportion of segments, across all respondents, with (a) capability greater than functioning, (b) capability equal to functioning, and (c) functioning greater than capability. Within those segments where capability exceeded functioning, the degree of divergence (i.e., one level, two levels, or three levels) was also calculated. Finally, the number of attributes with CFD (ranging from 0 to 5) was also calculated for each person who fully completed the instrument in the study.

The focus then turned to the degree to which CFDs were reported in different subgroups of respondents. To examine this, the mean capability level (ranging from 1 [*lowest*] to 4 [*highest*]), and the mean functioning level (ranging from 1 [*lowest*] to 4 [*highest*]), was estimated for each attribute. Subgroups were defined by age, sex, education, health, and informal care provision. Chi‐squared tests were used to infer whether CFD across each of the five attributes differed across subgroups. Statistical significance at the 5% level was highlighted.

Finally, CFDs were modelled as a function of the characteristics of the respondent to identify whether CFD was independently associated with the five subgrouping variables. Two variables were created to represent capability–functioning differences: (a) an indicator variable (CFDI), which took a value of 1 if a respondent reported any capability exceeding functioning on any attributes and 0 otherwise; and (b) an ordinal variable (CFDO), which took a value equal to the number of times capability exceeded functioning for each respondent. Both CFDI and CFDO were regressed on the same set of five dummy variables used to create the socio‐demographic subgroups reported earlier. These were sex (1 = male), age (1 = 50 years+), education (1 = degree), health status (HS, 1 = “full” health), and informal care (IC, 1 = informal care provided).

Model 1 CFDI = α + β_1_Sex + β_2_Age + β_3_Educ + β_4_HS + β_5_IC + μ
Model 2 CFDO = α + β_1_Sex + β_2_Age + β_3_Educ + β_4_HS + β_5_IC + μ

Both models were estimated at the respondent level where each observation related to a unique individual, rather than a segment. The regression analysis focused on modelling cases where capability exceeded functionings as the logical way in which capability and functioning would diverge. However, sensitivity analyses were conducted, including any cases where functioning was reported to exceed capability. All analyses were conducted in Stata MP v12.

## 3 RESULTS

### 3.1 Response to the survey

The response to the family impact of meningitis survey is described in more detail elsewhere (Al‐Janabi et al., 2016). When respondents were followed up 12 months later, 1,038 responses were received (gross response rate of 64%). During the process of data entry and cleaning, an additional 16 respondents were deemed ineligible, resulting in 1,022 useable responses (Table 1).

**Table 1:** Respondents to the follow‐up survey on the family impact of meningitis (*n* = 1,022)

Characteristic	Mean/frequency	Response
Age (mean, SD)	53.1 (12.6)	*n* = 1002, 98%
Sex		
Male	248 (25%)	*n* = 1,011, 99%
Female	763 (75%)	
Education (highest level of qualification)		
No qualifications	88 (9%)	*n* = 1,003, 99%
GCSEs or equivalent	246 (25%)	
A‐levels or equivalent	222 (22%)	
Degree or equivalent	447 (45%)	
Health status (mean [SD] EQ‐5D‐5L score)	0.86 (0.18)	*n* = 991, 97%
Caring responsibility		
Patient has no after‐effects	323^a^ (33%)	*n* = 987, 97%
Patient has after‐effects, but no informal care is provided	516 (52%)	
Patient has after‐effects, and informal is provided	148 (15%)	

a This includes four who report being an informal carer.

The response to the functioning and capability instrument is displayed in Table 2. The response rate to the functioning questions ranged from 93% (autonomy) to 96% (enjoyment), and the response rate to the capability questions ranged from 92% (enjoyment) to 93% (achievement and stability). Completion rates for capability questions, compared to functioning questions, were slightly lower across all attributes, although the differences were only significant (*p* < .05) in the case of attachment (*p* = .007) and enjoyment (*p* = .002). It is also worth noting that a larger proportion of people report the top level of capability as compared to the top level of functioning across all attributes. Consistent with this, a larger proportion of people report the bottom level of functioning, as compared to bottom level of capability, across all attributes.

**Table 2:** Response to the ICECAP‐FC (*n* = 1,022)

	Capability	Functioning
Autonomy	*n* = 949	*n* = 951
Completely independent	708 (75%)	665 (70%)
Independent in many things	200 (21%)	224 (24%)
Independent in a few things	36 (4%)	53 (6%)
Independent in no things	5 (1%)	9 (1%)
Achievement	*n* = 951	*n* = 959
Achieve and progress in all aspects of life	422 (44%)	340 (35%)
Achieve and progress in many aspects of life	416 (44%)	458 (48%)
Achieve and progress in a few aspects of life	100 (11%)	140 (15%)
Achieve and progress in no aspects of life	13 (1%)	21 (2%)
Stability	*n* = 951	*n* = 970
Settled and secure in all areas of life	416 (44%)	382 (39%)
Settled and secure in many areas of life	421 (44%)	437 (45%)
Settled and secure in a few areas of life	105 (11%)	135 (14%)
Settled and secure in no areas of life	9 (1%)	16 (2%)
Attachment	*n* = 946	*n* = 976
A lot of love, friendship, and support	696 (74%)	695 (71%)
Quite a lot of love, friendship, and support	198 (21%)	201 (21%)
A little love, friendship, and support	46 (5%)	73 (7%)
No love, friendship, and support	6 (1%)	7 (1%)
Enjoyment	*n* = 945	*n* = 979
A lot of enjoyment and pleasure	538 (57%)	486 (50%)
Quite a lot of enjoyment and pleasure	322 (44%)	355 (36%)
A little enjoyment and pleasure	80 (8%)	130 (13%)
No enjoyment and pleasure	5 (1%)	8 (1%)

### 3.2 Scale of divergence between capability and functioning

From 943 respondents, 4,637 completed capability–functioning segments were generated. These respondents comprised 895 who provided full data (generating 895 × 5 = 4,475 segments) and a further 48 who provided partial data (generating the remaining 162 segments).

Across all completed segments, capability exceeded functioning in 572 cases (12%), capability equalled functioning in 3,977 cases (86%), and functioning exceeded capability in 88 cases (2%). Logically, functioning should not exceed capability, and only a small proportion of people reported that it did so. The analysis of CFD in the in this section therefore focuses on the cases where capability exceeded functioning, with a sensitivity analysis conducted of the effect of including the segments where functioning was reported to exceed capability.

When capability exceeded functioning, there were 524 segments (92%) where functioning was one level below capability, there were 41 segments (7%) where functioning was two levels below capability, and there were 7 segments (1%) where functioning was three levels below capability. Across the sample providing complete capability and functioning data (*n* = 895), 590 (66%), people reported no CFD and 305 (34%) reported CFD in at least one attribute. This comprised 161 (18%) who reported CFD in one attribute, 74 (8%) who reported CFD in two attributes, and 70 (8%) who reported CFD in three or more attributes.

**Figure 3:**
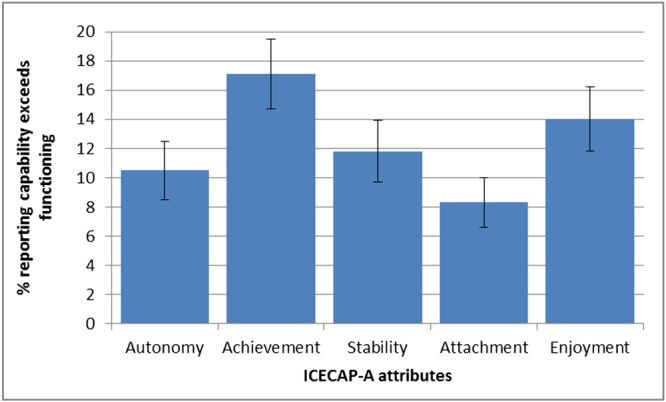
Proportion of individuals across each attribute reporting that capabilities exceeded functioning (*n* = 943). Error bars show 95% confidence intervals [Colour figure can be viewed at http://wileyonlinelibrary.com]

Figure 3 shows the proportion of CFD across each of the five attributes of the ICECAP‐FC. Respondents were most likely to report that their capability for “achievement” was greater than their functioning (17.1%) and least likely to report that their capability for “attachment” exceeded their functioning (8.3%). The proportion reporting CFD on achievement was significantly higher than the proportion reporting CFD on stability (95% CI [2.1%, 8.5%]), autonomy (95% CI, [3.6%, 10%]), or attachment (95% CI [5.9%, 11.9%]). The proportion reporting CFD on attachment was significantly lower than achievement (see above), enjoyment (−2.9% to −8.6%), and stability (−0.8% to −6.3%).

When responses where functioning was reported to exceed capability were included as CFD, 61% reported no CFD and 39% reported CFD in at least one attribute. The proportions reporting any CFD ranged from a high of 18.4% (achievement) to a low of 10.9% (attachment).

### 3.3 Capability–functioning differences by group

#### 3.3.1 Sex

**Figure 4:**
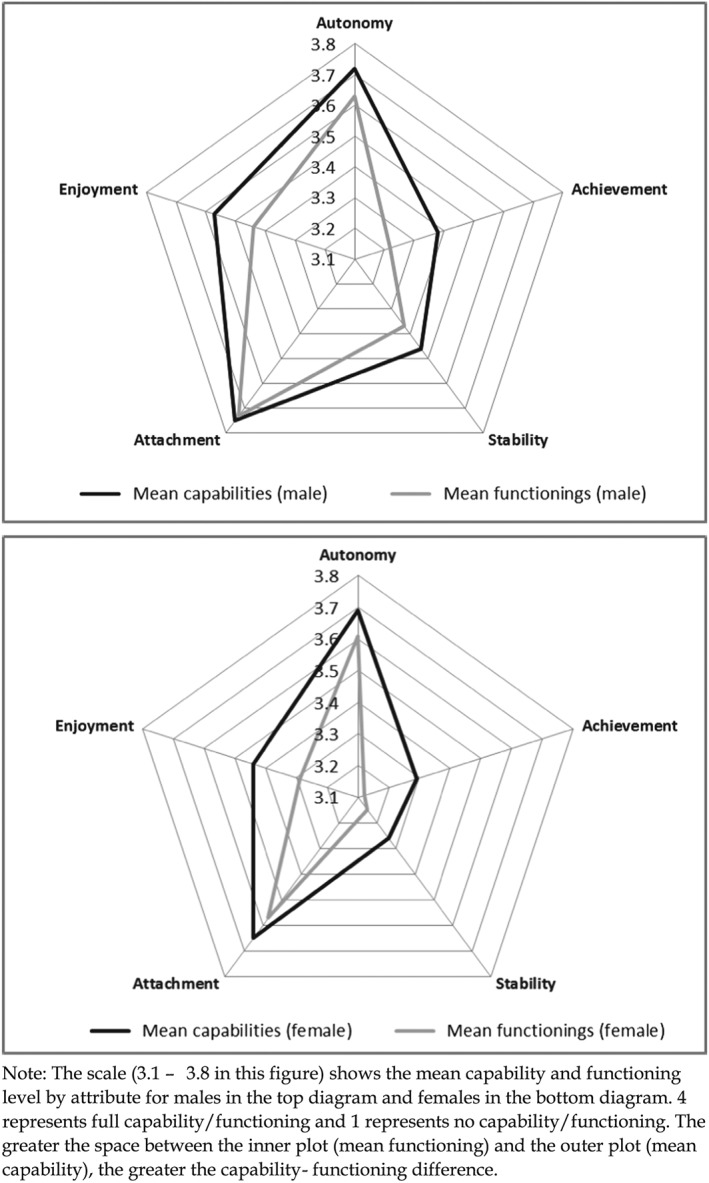
Mean capability set and functionings for males and females

Males tended to report higher levels of both capabilities *and* functionings than females, particularly in terms of enjoyment, achievement, and stability (Figure 4). However, females were more likely to report CFD, although this was significant only in the case of attachment where 9.8% of women reported CFD as opposed to 3.5% of males.

#### 3.3.2 Age

**Figure 5:**
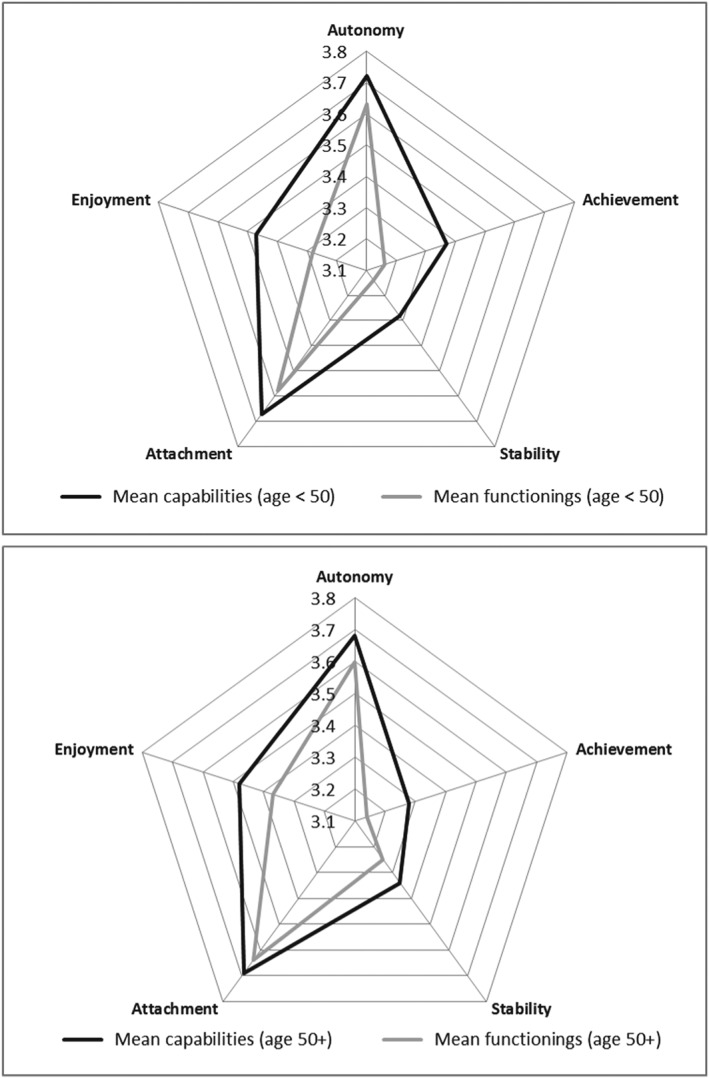
Mean capability set and functionings for those under 50 and over 50

Respondents were split into two groups, comprising those under 50 (*n* = 424) and those aged 50 and over (*n* = 580). Both groups reported similar levels of capabilities and functionings (Figure 5). However, across all attributes, there was a tendency for those aged 50 and over to be less likely to report CFD. This was significant in the case of achievement (20.9% vs. 14.3% vs. 20.9%), stability (9.9% vs. 14.5%), and enjoyment (11.5% vs. 17.4%).

#### 3.3.3 Education

**Figure 6:**
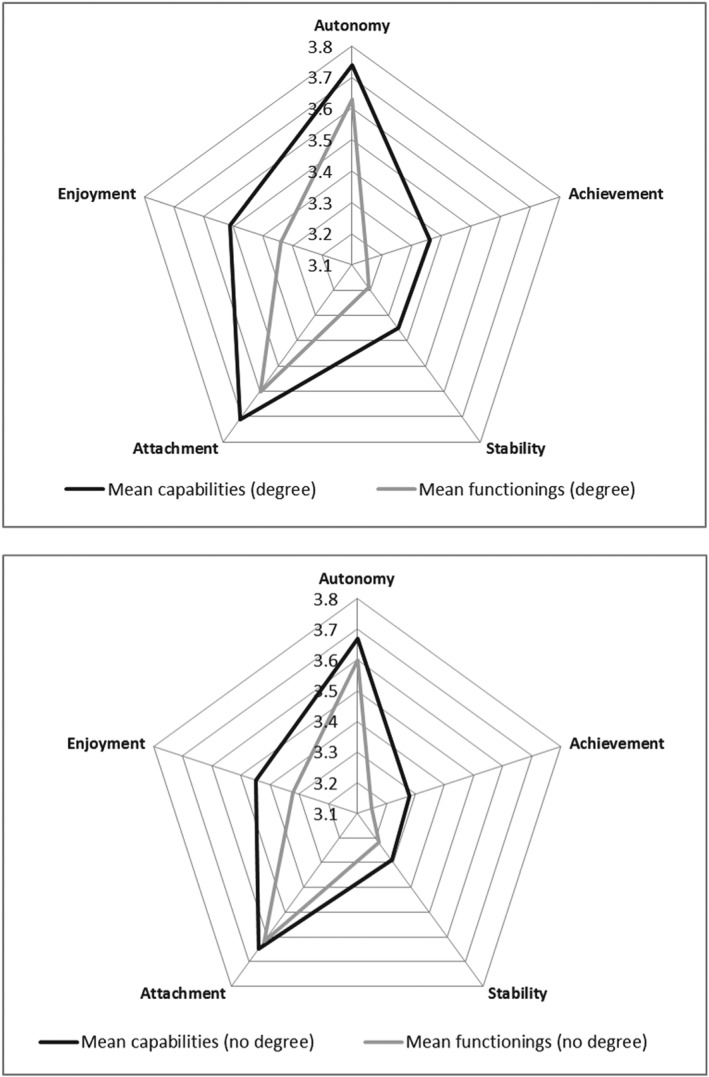
Mean capability set and functionings for those with and without a degree

Those with degree‐level education (*n* = 425) and those without (*n* = 504) reported very similar functionings, across all attributes (Figure 6). However, those with a degree tended to report higher capabilities. Reflecting this, those with a degree were more likely to report CFD. This was significant in the case of autonomy (13% vs. 7.7%), achievement (20.6% vs. 13.8%), stability (16.6% vs. 7.9%), and attachment (10.9% vs. 6%).

#### 3.3.4 Health status

**Figure 7:**
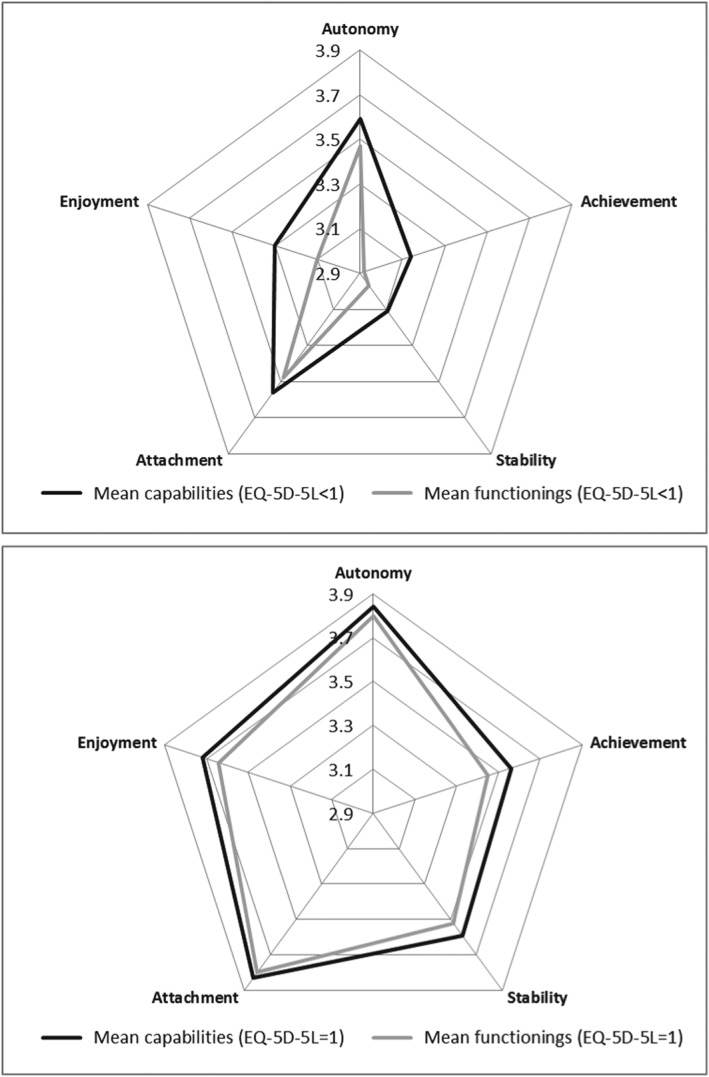
Mean capability set and functionings for with/without in full health

Those in full health (*n* = 400) reported higher levels of capability and functioning across all attributes than those people reporting impairment on one or more EQ‐5D‐5L domains (*n* = 532; Figure 7). Across all attributes, those in impaired health were more likely to report CFD. This was significant in the case of autonomy (14.7% vs. 5.4%), achievement (20.9% vs. 12.2%), stability (14.5% vs. 8.3%), attachment (11.4% vs. 3.5%), and enjoyment (18.6% vs. 7.8%).

#### 3.3.5 Informal care status

**Figure 8:**
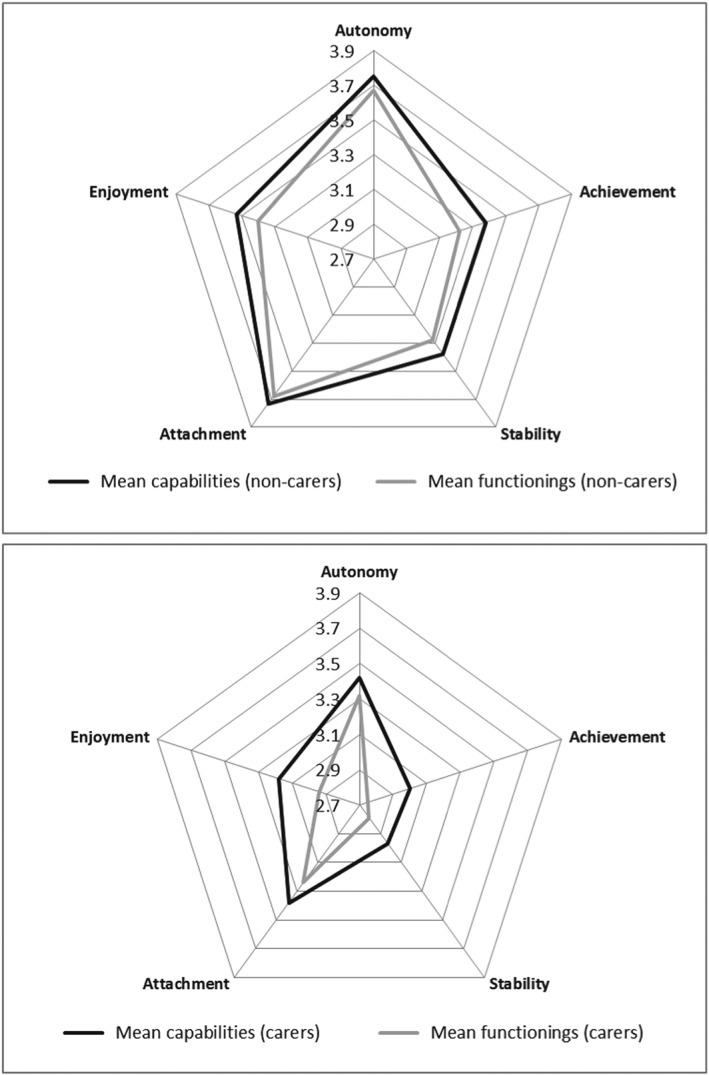
Mean capability set and functionings for noncarers and carers

People who did not report providing informal care reported higher levels of capability and functioning across all attributes (Figure 8). Across all attributes, except autonomy, those providing informal care were more likely to report CFD. This was significant in the case of achievement (23.8% vs. 15.9%), stability (17.1% vs. 10.9%), attachment (15.1% vs. 7%), and enjoyment (20.7% vs. 12.8%).

Table 3 shows the results of the multivariable regressions of CFD on the five contextual factors (age, sex, education, health status, and informal care). The presence of any CFD is studied in Model 1 and the number of capability–functioning differences is studied in Model 2. The two models provide very similar inferences, with both models indicating that degree‐level education and poor health status are the strongest independent predictors of CFD. The provision of informal care and being young is associated with a positive effect on CFD, although this is statistically significant only in Model 2. In the sensitivity analysis, which included responses where functionings were reported to exceed capability, degree‐level education and health status remained the strongest independent predictors of CFD. Additionally, in both Models 1 and 2 being under 50 and providing informal care were significant predictors of CFD (at the 5% level).

**Table 3:** Variables associated with capability–functioning differences

Variables	Model 1—(CFDI) Any capability–functioning differences	Model 2—(CFDO) Number of capability–functioning differences
Sex (male)	−0.11	−0.06
Education (degree)	0.60**	0.61**
Age (50+)	−0.25	−0.38*
Health status (EQ‐5D‐5L = 1)	−0.87**	−0.94**
Informal care (provided)	0.39	0.47*
Log likelihood	−527	−886
Pseudo‐*R* ^2^	0.05	0.04
Observations	871	871

* *p* < .05.

** *p* < .01.

## 4 DISCUSSION

This study examined the degree to which people report differences in their capabilities and functionings (CFD). Data collected using an amended version of the ICECAP‐A capability instrument indicates that around one third of people reported some difference in their capabilities and functionings and that they were most likely to do so in the context of achievement (17%) and least likely to do so in the context of attachment (8%). People with degree‐level education and impaired health status were more likely to report that their capability exceeded their functioning.

Response rates to the capability and functioning questions were quite high (>90%). This mirrors what others have found in terms of the feasibility of self‐report capability measures (Al‐Janabi, Peters, et al., 2013; Coast, Peters, Natarajan, Sproston, & Flynn, 2008). It also suggests that it is feasible to ask people to report on both their capability and functioning. Only a minority of responses (2%) were “illogical” in the sense that reported functionings *exceeded* reported capabilities. A greater propensity to report CFD in achievement, relative to attachment, was found in this study, and this is consistent with impressions formed following an earlier qualitative study (Al‐Janabi, Keeley et al., 2013). However, even in the case of attachment, around one in 12 people reported that their capability exceeded their functioning.

The finding that people with more education were more likely to report CFD would fit with a view that more empowered people are able to choose the life they want to live. These people may be more likely to trade‐off capabilities, explicitly choosing lower functionings in certain domains of life, resulting in more CFD. However, it may also be the case that people with a degree‐level education were more able to distinguish between the capability and functioning questions and therefore answer the two types of question differently.

It was also found that those in impaired health, or with caring responsibilities, were also more likely to report CFD. One explanation could be that carers and people in impaired health had past experiences of doing more in their lives. They may better understand what they are capable of, precisely because they know exactly what they did when they were healthy, or prior to providing informal care. Equally, they may be (or think they may be) more able to achieve higher functionings in the future. These respondents might essentially see their capabilities as innate and separate from their current circumstances. So while they currently feel limited in what they actually do in their lives, they perceive themselves to be capable of more achievement, stability, enjoyment, and so forth.

Another way of thinking about this is that this might be down to the perspective that respondents take. Some respondents may take a short‐term perspective focusing both on what they are innately capable of and what they are socially capable (given their current health, education, social circumstances, etc.). This seems to fit with Alkire's notion of a “combined capability” (Alkire, 2002). Conversely, some respondents may take a longer term perspective focusing on what they are innately capable of and viewing social circumstances as essentially variable in the longer term. This seems to fit with Alkire's notion of an “internal capability” (Alkire, 2002). Further work to explore the reasons for CFD, and the degree to which people respond in terms of internal or combined capability, could help to examine this issue further.

This study represents an initial investigation of the nature of differences in capabilities and functioning, and some limitations of this study are worth mentioning. First, data on capabilities were collected using a specific tool—the ICECAP‐A measure. The findings therefore relate to the domains of life covered by the ICECAP‐A measure, and different findings may emerge if other tools are used. Second, no information was collected on the reasons for CFD. This means that it is unclear which people reported CFD because they chose a lower functioning and which people reported CFD because they felt inhibited in their functionings by the circumstances of their life. These are areas where further quantitative and qualitative research would be helpful. Third, it may be the case that placing the capability and functioning questions alongside each drew more attention to the differences and the distinction would not have been so apparent if they had been separated. Future work could test this.

In general terms, both education and health are associated with higher capability and functioning. 1The relationship between higher education and functionings is non‐significant for most attributes. However, this study also implies that capability varied relatively more with education (Figure 6) whereas functioning varied relatively more with health status (Figure 7). This finding warrants further investigation—longitudinal data would be able to show whether health improvement is associated with more *change* in functioning as opposed to capability, for example. However, the findings do suggest that there may be a real difference in using one evaluative space vis‐à‐vis the other in evaluating public policy. Specifically investing in health care may be *more* attractive if the aim is to improve functionings and investing in education may be *more* attractive if the aim to improve capability.

In conclusion, this study suggests that a sizeable minority of people function below their level of capability. This is more likely in certain areas of life (achievement) and for certain people, such as those with degree‐level education or those in poor health. The findings provide some empirical support for the notion that capability data capture something in addition to functioning data and indeed that the choice of evaluative space may influence the relative priorities within the public sector.

## CONFLICTS OF INTEREST

The author is the codeveloper of the ICECAP‐A capability measure and is not aware of any financial or personal relationships that might be perceived as biasing the work reported here.

## References

[hec3586-bib-0001] Al‐Janabi, H. , Flynn, T. , & Coast, J. (2012). Development of a self‐report measure of capability wellbeing for adults: The ICECAP‐A. Quality of Life Research, 21, 167–176. 21598064 10.1007/s11136-011-9927-2 PMC3254872

[hec3586-bib-0002] Al‐Janabi, H. , Flynn, T. , Peters, T. , Bryan, S. , & Coast, J. (2015). Test‐retest reliability of capability measurement in the UK population. Health Economics, 24, 625–630. 25204621 10.1002/hec.3100 PMC4405059

[hec3586-bib-0003] Al‐Janabi, H. , Keeley, T. , Mitchell, P. , & Coast, J. (2013). Can capabilities be self‐reported? A think‐aloud study. Social Science and Medicine, 86, 116–122. 10.1016/j.socscimed.2013.03.035 PMC3664929 23631786

[hec3586-bib-0004] Al‐Janabi, H. , Peters, T. , Brazier, J. , Bryan, S. , Flynn, T. , Clemens, S. , … Coast, J. (2013). An investigation of the construct validity of the ICECAP‐A capability measure. Quality of Life Research, 22, 1831–1840. 23086535 10.1007/s11136-012-0293-5 PMC3764327

[hec3586-bib-0005] Al‐Janabi, H. , Van Exel, J. , Brouwer, W. , Trotter, C. , Glennie, L. , Hannigan, L. , & Coast, J. (2016). Measuring health spillovers for economic evaluation: A case study in meningitis. Health Economics, 25, 1529–1544. 26464311 10.1002/hec.3259 PMC5111598

[hec3586-bib-0006] Alkire, S. (2002). Valuing freedoms. Oxford: Oxford University Press.

[hec3586-bib-0007] Anand, P. (2005). Capabilities and health. Journal of Medical Ethics, 31, 299–303. 15863692 10.1136/jme.2004.008706 PMC1734139

[hec3586-bib-0008] Anand, P. , Hunter, G. , Carter, I. , Dowding, K. , Guala, F. , & Van Hees, M. (2009). The development of capability indicators. Journal of Human Development and Capabilities, 10, 125–152.

[hec3586-bib-0009] Birch, S. , & Donaldson, C. (2003). Valuing the benefits and costs of health care programmes: Where's the ‘extra’ in extra‐welfarism? Social Science and Medicine, 56, 1121–1133. 12593883 10.1016/s0277-9536(02)00101-6

[hec3586-bib-0010] Bleichrodt, H. , & Quiggin, J. (2013). Capabilities as menus: A non‐welfarist basis for QALY evaluation. Journal of Health Economics, 32, 128–137. 23202258 10.1016/j.jhealeco.2012.10.004

[hec3586-bib-0011] Brazier, J. , Ratcliffe, J. , Salomon, J. , & Tsuchiya, A. (2007). Measuring and valuing health benefits for economic evaluation. Oxford: Oxford University Press.

[hec3586-bib-0012] Brouwer, W. , Culyer, A. , Van Exel, J. , & Rutten, F. (2008). Welfarism vs extra‐welfarism. Journal of Health Economics, 27, 325–338. 18179835 10.1016/j.jhealeco.2007.07.003

[hec3586-bib-0013] Burge, P. , Netten, A. , & Gallo, F. (2010). Estimating the value of social care. Journal of Health Economics, 29, 883–894. 20863584 10.1016/j.jhealeco.2010.08.006

[hec3586-bib-0014] Canaway, A. , Al‐Janabi, H. , Kinghorn, P. , Bailey, C. , & Coast, J. (2017). Development of a measure (ICECAP‐Close Person Measure) through qualitative methods to capture the benefits of end‐of‐life care to those close to the dying for use in economic evaluation. Palliative Medicine, 31, 53–62. 27260168 10.1177/0269216316650616

[hec3586-bib-0015] Chiappero‐Martinetti, E. , & Roche, J.‐M. (2009). Operationalization of the capability approach, from theory to practice: A review of techniques and empirical applications In Chiappero‐Martinetti E. (Ed.), Debating global society: Reach and limits of the capability approach. Milan: Fondazione Giangiacomo Feltrinelli.

[hec3586-bib-0016] Coast, J. , Flynn, T. , Natarajan, L. , Sproston, K. , Lewis, J. , Louviere, J. , & Peters, T. (2008). Valuing the ICECAP capability index for older people. Social Science and Medicine, 67, 874–882. 18572295 10.1016/j.socscimed.2008.05.015

[hec3586-bib-0017] Coast, J. , Peters, T. , Natarajan, L. , Sproston, K. , & Flynn, T. (2008). An assessment of the construct validity of the descriptive system for the ICECAP capability measure for older people. Quality of Life Research, 17, 967–976. 18622721 10.1007/s11136-008-9372-z

[hec3586-bib-0018] Coast, J. , Smith, R. , & Lorgelly, P. (2008). Should the capability approach be applied in health economics. Health Economics, 17, 667–670. 18457341 10.1002/hec.1359

[hec3586-bib-0019] Cookson, R. (2005). QALYs and the capability approach. Health Economics, 14, 817–829. 15693028 10.1002/hec.975

[hec3586-bib-0020] Gasper, D. (2007). What is the capability approach? It's core, rationale, partners and dangers. Journal of Socio‐Economics, 36, 335–359.

[hec3586-bib-0021] Goranitis, I. , Coast, J. , Al‐Janabi, H. , Latthe, P. & Roberts, T. (2016). The validity and responsiveness of the ICECAP‐A capability‐well‐being measure in women with irritative lower urinary tract symptoms. *Quality of Life Research*, In press, 1‐13. 10.1007/s11136-015-1225-y PMC4945699 26754141

[hec3586-bib-0022] Greco, G. , Skordis‐Worrall, J. , Mkandawire, B. , & Mills, A. (2015). What is a good life? Selecting capabilities to assess women's quality of life in rural Malawi. Social Science & Medicine, 130, 69–78. 25687242 10.1016/j.socscimed.2015.01.042

[hec3586-bib-0023] Grewal, I. , Lewis, J. , Flynn, T. , Brown, J. , Bond, J. , & Coast, J. (2006). Developing attributes for a generic quality of life measure for older people: Preferences or capabilities? Social Science and Medicine, 62, 1891–1901. 16168542 10.1016/j.socscimed.2005.08.023

[hec3586-bib-0024] Herdman, M. , Gudex, C. , Lloyd, A. , Janssen, M. , Kind, P. , Parkin, D. , … Badia, X. (2011). Development and preliminary testing of the new five‐level version of the EQ‐5D (EQ‐5D‐5L). Quality of Life Research, 20, 1727–1736. 21479777 10.1007/s11136-011-9903-x PMC3220807

[hec3586-bib-0025] Kahneman, D. , & Sugden, R. (2005). Experienced utility as a standard of policy evaluation. Environmental & Resource Economics, 32, 161–181.

[hec3586-bib-0026] Keeley, T. , Al‐Janabi, H. , Nicholls, E. , Foster, N. , Jowett, S. , & Coast, J. (2015). A longitudinal assessment of the responsiveness of the ICECAP‐A in a randomised controlled trial of a knee pain intervention. Quality of Life Research, 24, 2319–2331. 25894061 10.1007/s11136-015-0980-0 PMC4564441

[hec3586-bib-0027] Kinghorn, P. , Robinson, A. , & Smith, R. (2015). Developing a capability‐based questionnaire for assessing well‐being in patients with chronic pain. Social Indicators Research, 120, 897–916.

[hec3586-bib-0028] Lorgelly, P. , Lorimer, K. , Fenwick, E. , Briggs, A. , & Anand, P. (2015). Operationalising the capability approach as an outcome measure in public health: The development of the OCAP‐18. Social Science & Medicine, 142, 68–81. 26291444 10.1016/j.socscimed.2015.08.002

[hec3586-bib-0029] Malley, J. , Towers, A.‐M. , Netten, A. , Brazier, J. , Forder, J. , & Flynn, T. (2012). An assessment of the construct validity of the ASCOT measure of social care‐related quality of life with older people. Health and Quality of Life Outcomes, 10. 22325334 10.1186/1477-7525-10-21 PMC3305488

[hec3586-bib-0030] Mitchell, P. , Al‐Janabi, H. , Richardson, J. , Iezzi, A. , & Coast, J. (2015). The relative impacts of disease on health status and capability wellbeing: A multi‐country study. PloS One, 10. e0143590 10.1371/journal.pone.0143590 PMC4667875 26630131

[hec3586-bib-0031] NICE . 2013 Guide to the methods of technology appraisal (PMG9). Retrieved from NICE website: https://www.nice.org.uk/process/pmg9/chapter/foreword

[hec3586-bib-0032] Nussbaum, M. (2000). Women and human development: The capabilities approach. New York: Cambridge University Press.

[hec3586-bib-0033] Robeyns, I. (2005). The capability approach: A theoretical survey. Journal of Human Development, 6, 93–114.

[hec3586-bib-0034] Ryan, M. , Netten, A. , Skatun, D. , & Smith, P. (2006). Using discrete choice experiments to estimate a preference‐based measure of outcome—An application to social care for older people. Journal of Health Economics, 25, 927–954. 16464513 10.1016/j.jhealeco.2006.01.001

[hec3586-bib-0035] Sen, A. (1993). Capability and well‐being In Nussbaum M., & Sen A. (Eds.), The quality of life. Oxford: Oxford University Press.

[hec3586-bib-0036] Sen, A. (2009). The idea of justice. London: Allen Lane.

[hec3586-bib-0037] Simon, J. , Anand, P. , Gray, A. , Rugkåsa, J. , Yeeles, K. , & Burns, T. (2013). Operationalising the capability approach for outcome measurement in mental health research. Social Science and Medicine, 98, 187–196. 24331898 10.1016/j.socscimed.2013.09.019

[hec3586-bib-0038] Sugden, R. (2003). Opportunity as a space for individuality: Its value and the impossibility of measuring it. Ethics, 113, 783–809.

[hec3586-bib-0039] Sutton, E. , & Coast, J. (2014). Development of a supportive care measure for economic evaluation of end‐of‐life care using qualitative methods. Palliative Medicine, 28, 151–157. 23698452 10.1177/0269216313489368

[hec3586-bib-0040] Van Hout, B. , Janssen, M. , Feng, Y. , Kohlmann, T. , Busschbach, J. , Golicki, D. , … Pickard, S. (2012). Interim scoring for the EQ‐5D‐5L: Mapping the EQ‐5D‐5L to EQ‐5D‐3L value sets. Value in Health, 15, 708–715. 22867780 10.1016/j.jval.2012.02.008

